# Imaging–Endoscopy Discordance in Occult Tonsillar Tumors: Diagnostic Challenges of Submucosal Mucoepidermoid Carcinoma—A Case Report and Literature Review

**DOI:** 10.3390/diagnostics16182938

**Published:** 2026-09-11

**Authors:** Jia-Hua Yen, Ming-Che Ou, Jong-Shiaw Jin, Yi-Ching Chen, Tzu-Pin Lu, Hao-Chun Hung, Chun-Hsiang Chang

**Affiliations:** 1 Department of Radiation Oncology, Tungs’ Taichung MetroHarbor Hospital, Taichung 435, Taiwan; fluttery1991@gmail.com; 2 Institute of Epidemiology and Preventive Medicine, National Taiwan University, Taipei 100, Taiwan; tplu@ntu.edu.tw; 3 Department of Hematology, Tungs’ Taichung MetroHarbor Hospital, Taichung 435, Taiwan; t10247@ms3.sltung.com.tw; 4 Department of Pathology, Tungs’ Taichung MetroHarbor Hospital, Taichung 435, Taiwan; t10121@ms3.sltung.com.tw; 5 Department of Medical Research, Tungs’ Taichung MetroHarbor Hospital, Taichung 435, Taiwan; cf0817@gmail.com; 6 Institute of Health Data Analytics and Statistics, National Taiwan University, Taipei 100, Taiwan; 7 Department of Diagnostic Radiology, Tungs’ Taichung MetroHarbor Hospital, Taichung 435, Taiwan; 8 Department of Otolaryngology, Tungs’ Taichung MetroHarbor Hospital, Taichung 435, Taiwan

**Keywords:** mucoepidermoid carcinoma, p16 expression, occult primary tumor, imaging–endoscopy discordance, palatine tonsil

## Abstract

**Background and Clinical Significance:** Cervical lymphadenopathy with an occult primary tumor represents a common yet diagnostically challenging clinical scenario in head and neck oncology. While endoscopic evaluation is typically relied upon for tumor localization, submucosal lesions may remain undetected. **Case Presentation:** We report a 41-year-old woman presenting with an isolated right level II neck mass without constitutional symptoms. Initial fiberscopic examination revealed no mucosal abnormalities; however, contrast-enhanced computed tomography demonstrated asymmetric enlargement of the right palatine tonsil with effacement of the adjacent parapharyngeal fat plane, alongside multiple enlarged cervical lymph nodes. Subsequent positron emission tomography supported focal metabolic activity in the tonsillar region. Histopathological examination confirmed a low-grade mucoepidermoid carcinoma arising from the minor salivary glands of the palatine tonsil. Immunohistochemistry showed strong p16 positivity (70%), while MAML2 rearrangement was not detected. **Conclusions:** This case highlights a critical pitfall where imaging–endoscopy discordance signals a submucosal malignancy. Furthermore, strong p16 expression should be interpreted in conjunction with tumor morphology and clinical context, as p16 positivity is not entirely specific for HPV-related squamous cell carcinoma. Through integration of the current case with a focused literature review, we propose a structured diagnostic framework emphasizing imaging prioritization and morphology-based interpretation to improve recognition of occult submucosal tonsillar malignancies.

## 1. Introduction

Cervical lymphadenopathy with an occult primary tumor is a well-recognized diagnostic challenge in head and neck oncology [1,2]. In clinical practice, evaluation typically relies on a combination of physical examination and endoscopic assessment to identify mucosal lesions within the upper aerodigestive tract. However, this approach may fail when tumors arise from submucosal structures or exhibit minimal surface change, resulting in negative endoscopic findings despite the presence of malignancy [1,2].

In such scenarios, cross-sectional imaging plays a pivotal role in detecting subtle abnormalities that are not apparent on endoscopy [2,3]. In particular, asymmetric tonsillar enlargement and obliteration of the adjacent parapharyngeal fat plane may indicate an underlying infiltrative process rather than benign asymmetry [3,4,5]. Recognition of this imaging–endoscopy discordance is critical, as it may represent the only clue to an otherwise occult primary tumor [2,4,5,6].

Mucoepidermoid carcinoma (MEC) is the most common malignant tumor of the salivary glands [7,8], but involvement of the palatine tonsil is exceptionally uncommon [9,10,11,12,13]. The rarity of palatine tonsillar MEC is also reflected in the limited historical literature. When Jarvis et al. reported their case in 2013, only one previous case had been identified in the literature; by 2017, Karkuzhali and Sahoo reported that only three cases had been documented [10,11]. Due to its uncommon location and frequent submucosal growth pattern, tonsillar MEC may evade detection on routine endoscopic examination and be misinterpreted as metastatic disease of unknown primary origin [9,10,11,12,13].

In this report, we present a case of occult tonsillar MEC manifesting as isolated cervical lymphadenopathy with negative endoscopic findings. This case highlights the diagnostic challenge of recognizing submucosal tumors and interpreting p16 expression in the appropriate morphologic context, emphasizing the importance of integrating imaging, endoscopy, and histopathology. We further propose a structured diagnostic framework based on imaging–endoscopy discordance to facilitate accurate tumor localization and avoid misclassification in similar clinical settings.

## 2. Case Presentation

A 41-year-old woman presented to Tungs’ Taichung MetroHarbor Hospital on 19 September 2024, with a painless right-sided neck mass that had been progressively enlarging over several months. She denied constitutional symptoms, including weight loss, fever, or night sweats, and reported no sore throat, dysphagia, hoarseness, or oral ulceration. Her medical history was unremarkable, with no history of smoking, alcohol consumption, or betel nut use. She had no recent dental procedures or significant dental history.

Physical examination revealed a firm, mobile, non-tender lymph node measuring approximately 2–3 cm in the right level II region. Examination of the oral cavity and oropharynx showed intact mucosa without visible lesions. Flexible nasopharyngolaryngoscopy demonstrated no abnormalities in the nasopharynx, oropharynx, or hypopharynx.

**Figure 1:**
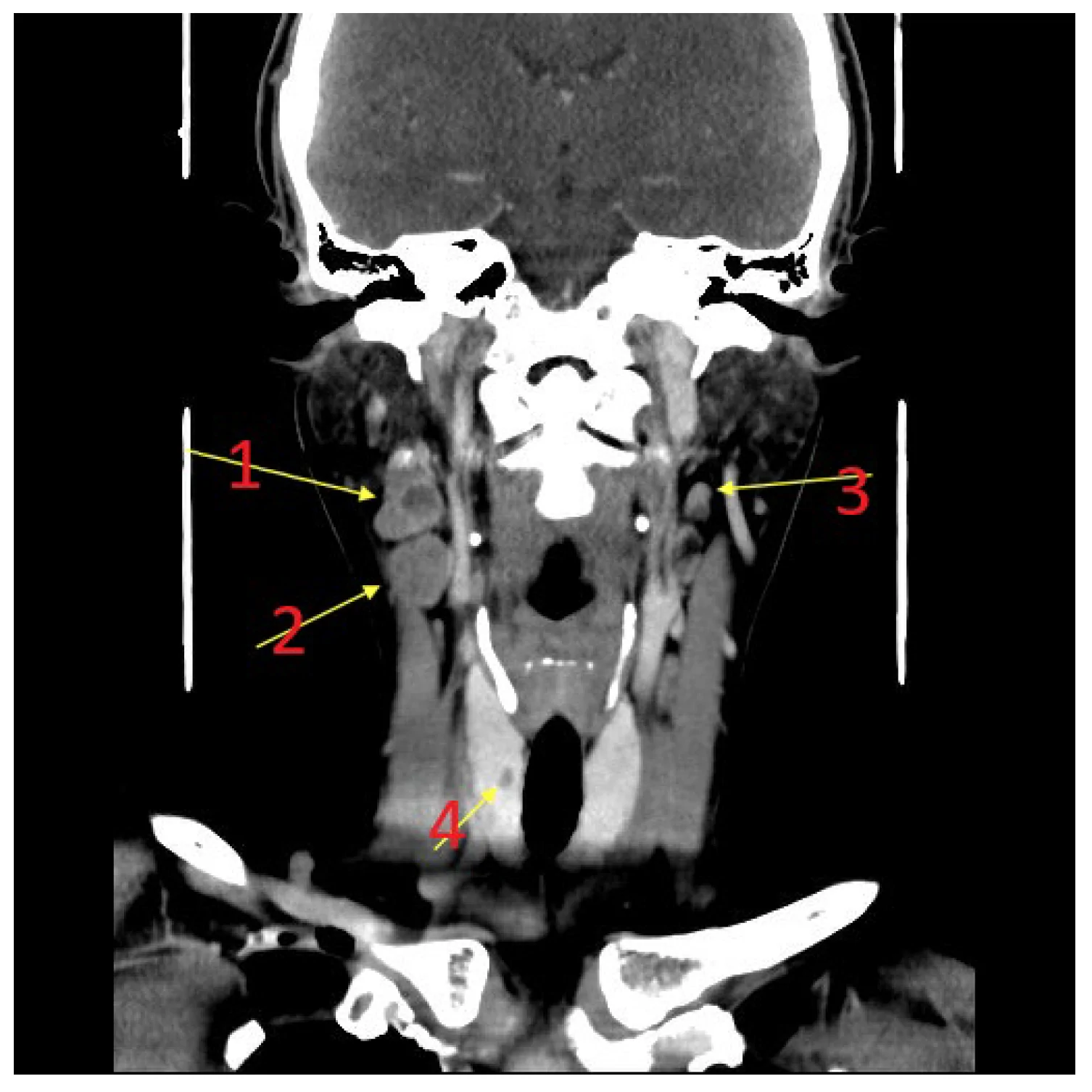
Contrast-enhanced coronal CT of the neck demonstrating enlarged cervical lymph nodes in the right level II (arrow 1), right level III (arrow 2), and left level II (arrow 3) regions. Arrow 4 indicates a small incidental thyroid nodule.

Given the clinical suspicion of cervical lymphadenopathy of unknown primary origin, contrast-enhanced computed tomography (CT) of the neck was performed. Imaging revealed enlarged cervical lymph nodes involving the right level II and III regions and the left level II region (Figure 1).

**Figure 2:**
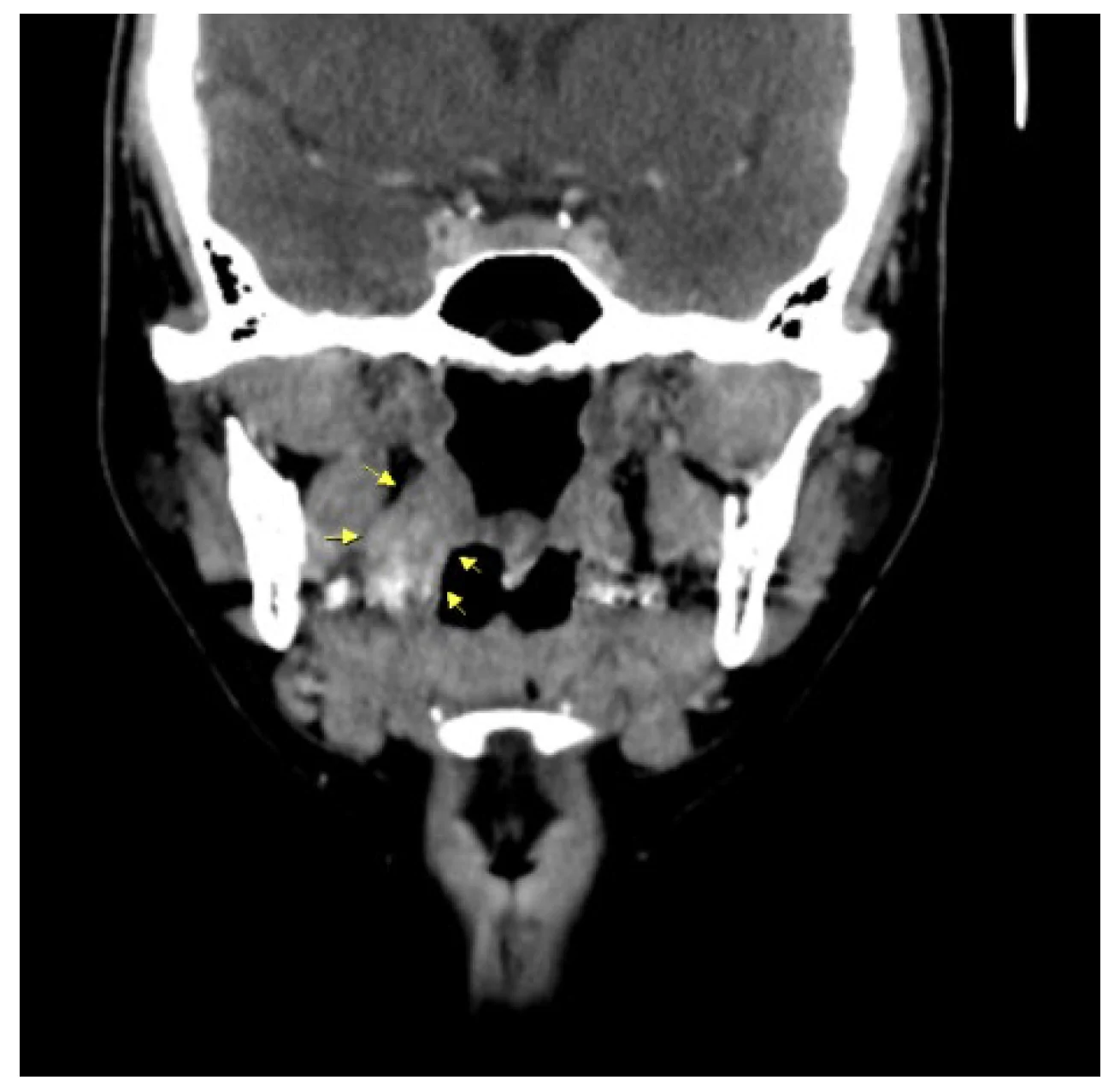
Contrast-enhanced CT showing asymmetric enlargement of the right palatine tonsil with effacement of the adjacent parapharyngeal fat plane, suggestive of a submucosal lesion.

In addition, careful review of the oropharynx demonstrated subtle asymmetric enlargement of the right palatine tonsil with effacement of the adjacent parapharyngeal fat plane, raising suspicion for a deep-seated tonsillar lesion despite the absence of mucosal abnormalities on endoscopy (Figure 2).

**Figure 3:**
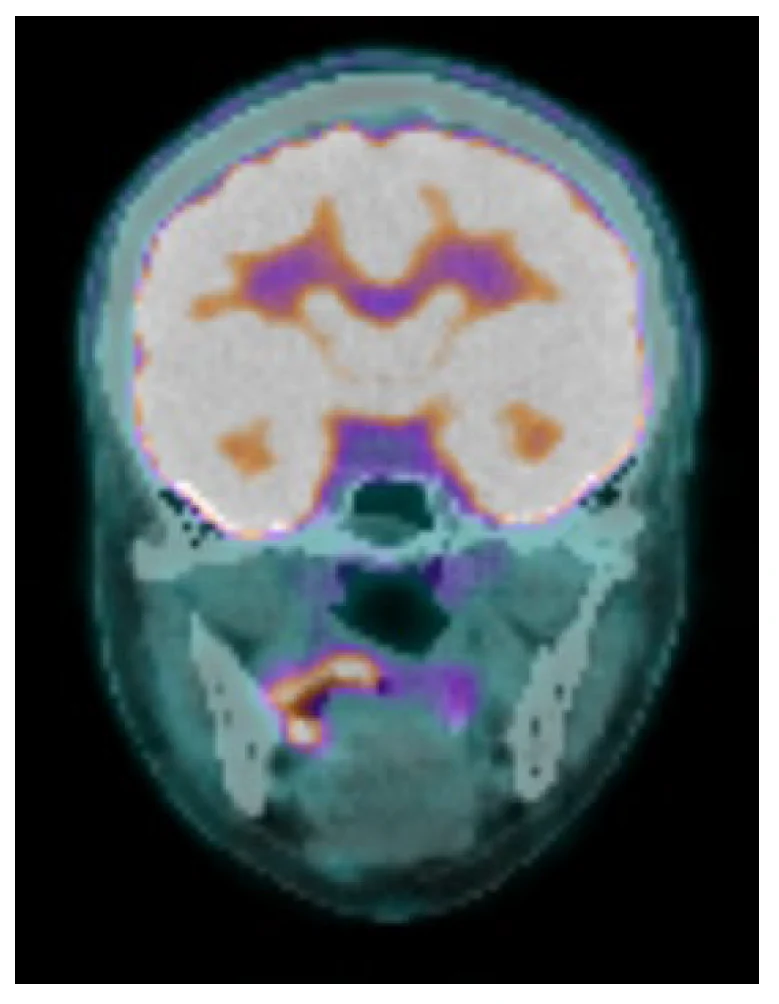
Coronal fluorodeoxyglucose (FDG) positron emission tomography–computed tomography (PET–CT) demonstrating focal increased metabolic activity in the right tonsillar region, corresponding to the suspected primary site, with associated metabolically active cervical lymphadenopathy.

To further evaluate the suspected primary site, positron emission tomography–computed tomography (PET–CT) was performed and demonstrated focal increased metabolic activity in the right tonsillar region, corresponding to the area of asymmetry identified on CT (Figure 3). No distant metastases were detected. Systemic evaluation included PET–CT, head-and-neck CT, and chest radiography, with no additional primary malignancy identified.

Based on the cervical nodal disease and suspicious right tonsillar imaging findings despite negative endoscopy, an occult tonsillar carcinoma was clinically suspected. The patient subsequently underwent transoral radical tonsillectomy and right level II neck dissection. Gross examination of the resected tonsillar specimen revealed a well-circumscribed tumor measuring 3 × 2 cm located within the tonsillar parenchyma.

**Figure 4:**
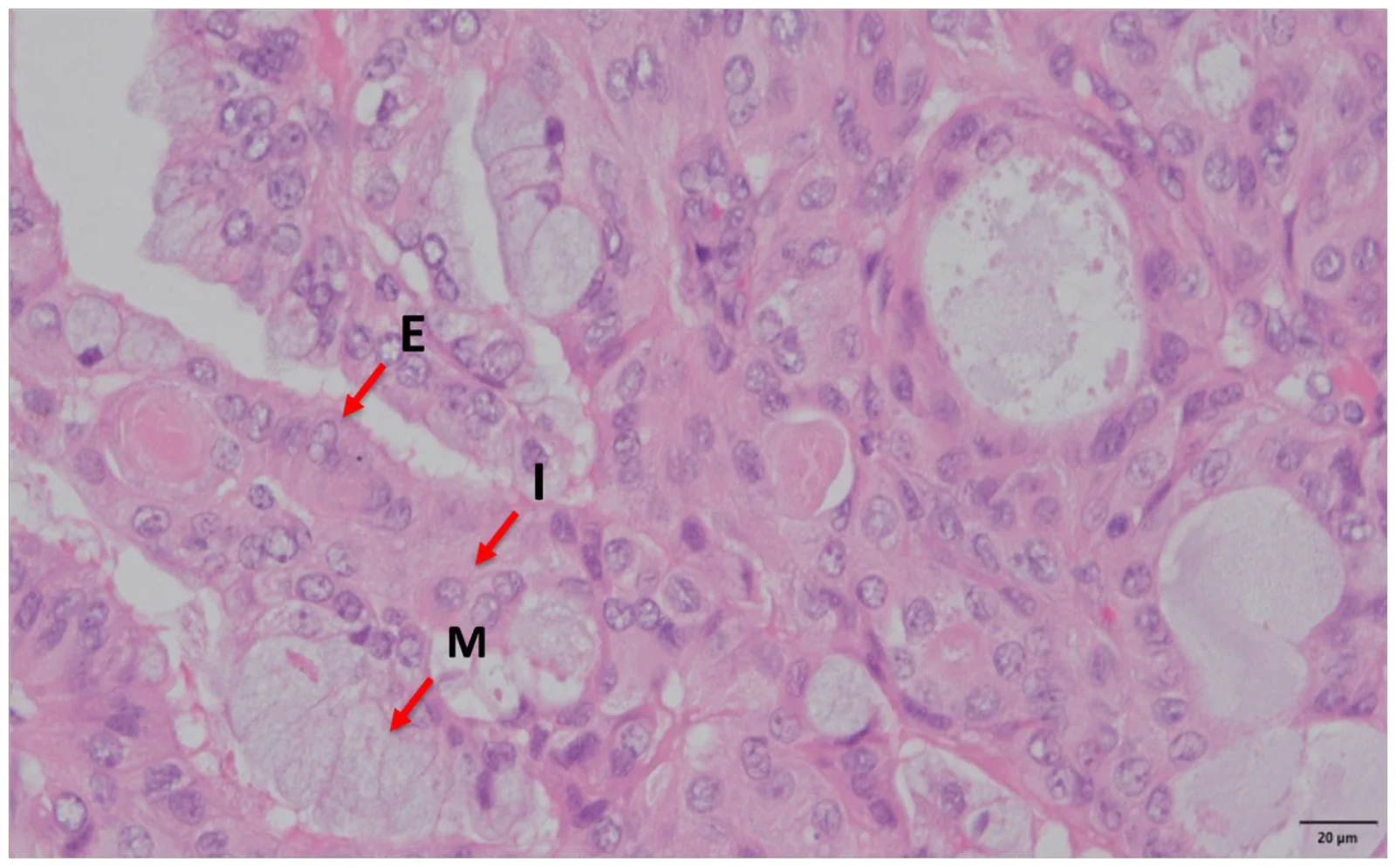
Histopathological examination showing the characteristic cellular components of mucoepidermoid carcinoma. Representative epidermoid (E), intermediate (I), and mucous (M) cells are indicated by red arrows. Hematoxylin and eosin (H&E) staining; original magnification, ×400; scale bar = 20 μm.

Histopathological examination showed a neoplasm composed of mucous cells, intermediate cells, and epidermoid cells arranged in cystic, papillary, and solid patterns, consistent with mucoepidermoid carcinoma (Figure 4).

According to Armed Forces Institute of Pathology (AFIP) grading criteria, the tumor was classified as low-grade, with no significant necrosis, neural invasion, high mitotic activity, or marked atypia.

Two of six dissected lymph nodes were positive for metastatic carcinoma, with extranodal extension identified. Surgical margins were free but close (2–5 mm).

**Figure 5:**
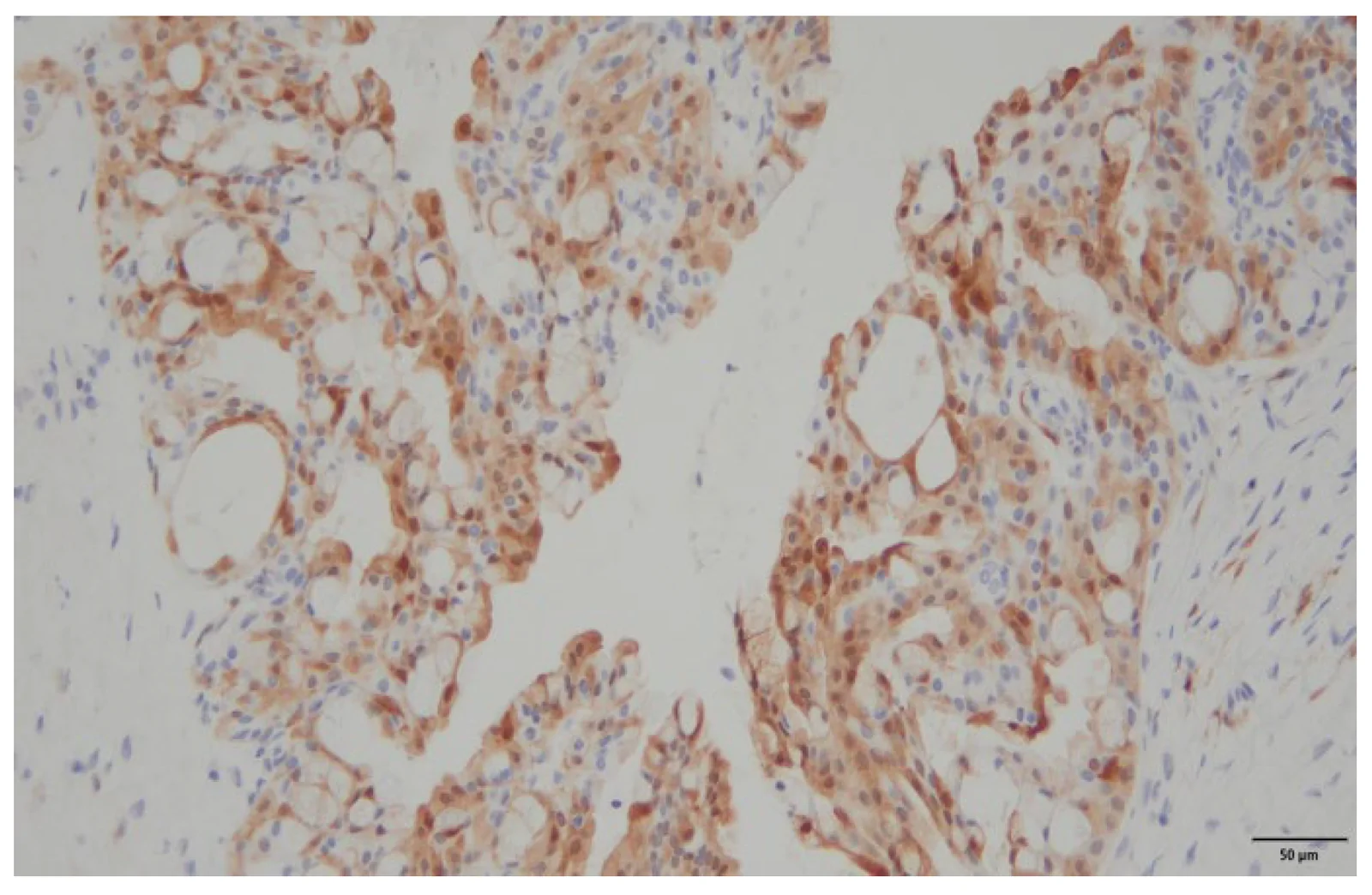
Immunohistochemical staining for p16 showing strong diffuse nuclear and cytoplasmic positivity in tumor cells.

Immunohistochemical staining demonstrated strong diffuse p16 positivity in approximately 70% of the tumor cells (Figure 5). Molecular analysis for MAML2 rearrangement was performed but did not detect the characteristic translocation.

Based on the histopathological and clinical findings, the final diagnosis was low-grade mucoepidermoid carcinoma arising from the minor salivary glands of the right palatine tonsil, with regional lymph node metastasis and extranodal extension.

The case was discussed in a multidisciplinary tumor board, and given the presence of high-risk features, including extranodal extension and close surgical margins, postoperative concurrent chemoradiotherapy was recommended.

The patient received intensity-modulated radiotherapy (66 Gy in 33 fractions) targeting the primary tumor bed and bilateral neck, with concurrent weekly cisplatin, from 13 November to 27 December 2024, over a total treatment duration of 44 days.

**Figure 6:**
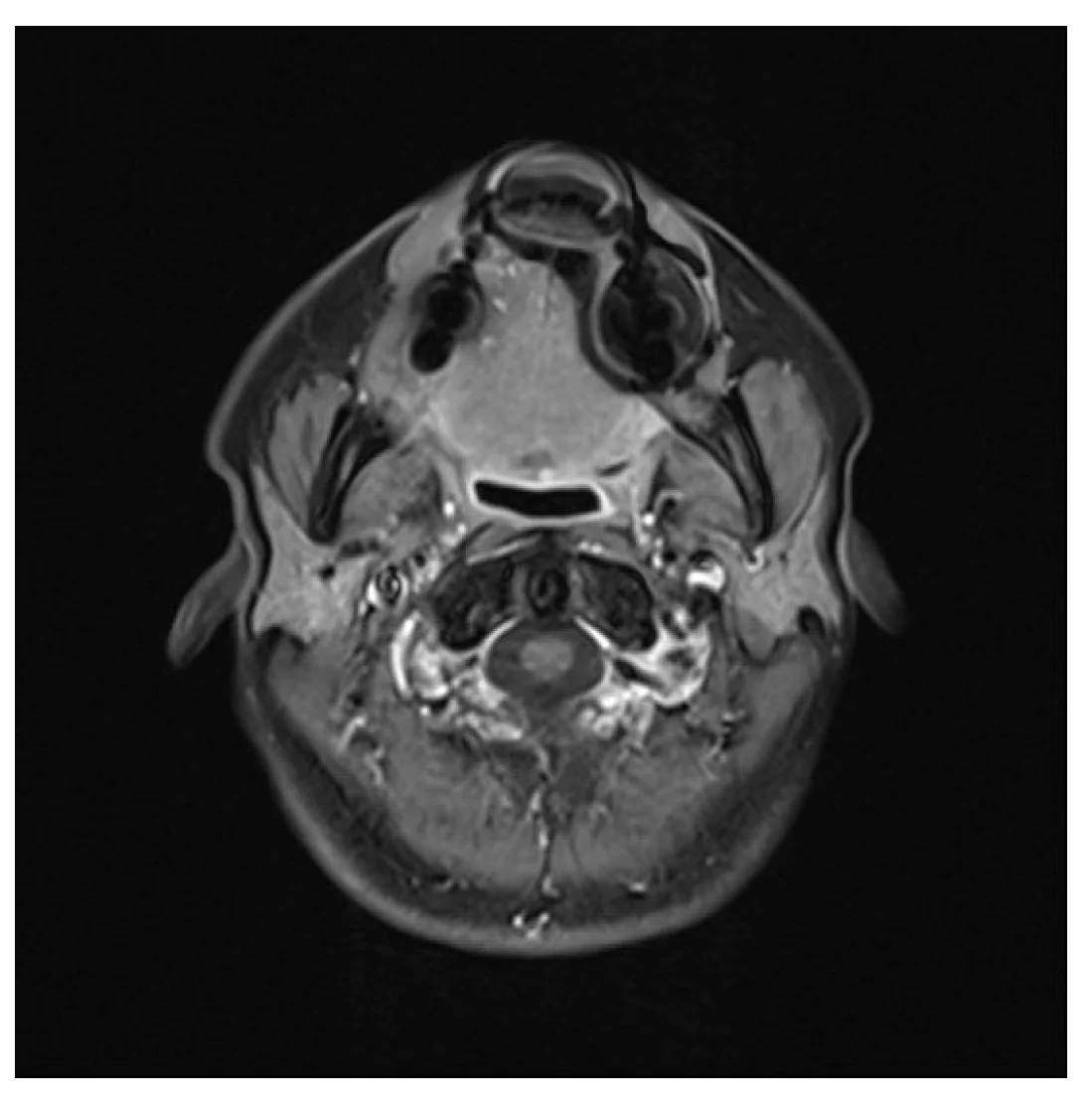
Follow-up magnetic resonance imaging (MRI) performed approximately 10 months after completion of radiotherapy, demonstrating regression of cervical lymphadenopathy with no obvious evidence of tumor recurrence.

Follow-up magnetic resonance imaging (MRI) on 18 October 2025, approximately 10 months after completion of radiotherapy, demonstrated regression of the cervical lymphadenopathy with no obvious tumor recurrence (Figure 6). At the latest outpatient follow-up on 30 April 2026, the patient remained alive without clinical evidence of local recurrence or cervical lymphadenopathy.

## 3. Discussion

Occult primary tumors presenting with cervical lymph node metastasis remain a diagnostic challenge in head and neck oncology [1,2]. Although physical examination and flexible endoscopy are routinely used to localize mucosal lesions within the upper aerodigestive tract, submucosal tumors or lesions with minimal surface alteration may escape direct visualization [1,2]. In such cases, imaging becomes essential for identifying abnormalities that are not clinically apparent. In our patient, the primary tonsillar lesion was undetectable on initial endoscopic examination despite metastatic cervical lymphadenopathy.

Imaging–endoscopy discordance may therefore provide an important clue to an occult primary tumor [2,4,5,6]. Asymmetric tonsillar enlargement, focal enhancement, and obliteration of the adjacent parapharyngeal fat plane on contrast-enhanced CT may indicate an infiltrative submucosal process despite normal mucosal findings [3,4,5]. In the present case, CT demonstrated subtle right tonsillar asymmetry with effacement of the surrounding fat plane, while PET–CT showed corresponding focal hypermetabolic activity. These findings prompted targeted evaluation of the tonsil despite negative endoscopy. Thus, persistent radiologic abnormalities should not be disregarded solely because mucosal examination is unremarkable.

Mucoepidermoid carcinoma (MEC) is the most common malignant tumor of the salivary glands [7,8], but is rarely encountered in the palatine tonsil [9,10,11,12,13]. Tonsillar MEC is believed to arise from minor salivary glands within tonsillar tissue and may exhibit predominantly submucosal growth [7,8]. Published cases are summarized in Table 1. Clinical manifestations have been variable, and occult or submucosal presentations may complicate early diagnosis [9,10,11,12,13]. Compared with previously reported cases, the present case demonstrates three notable features: the primary lesion remained occult on flexible endoscopy despite suspicious CT and PET–CT findings; the tumor showed strong diffuse p16 positivity requiring interpretation in the context of its morphology, and low-grade histology coexisted with nodal metastasis and extranodal extension. Although the primary tumor fulfilled histopathologic criteria for low-grade MEC, the presence of regional nodal metastasis and extranodal extension represented adverse clinical and pathologic features. This discordance suggests that histologic grade alone may not fully reflect clinical behavior and underscores the importance of incorporating nodal status and other adverse pathologic features into risk assessment and treatment planning. These findings extend the reported spectrum of tonsillar MEC and reinforce the value of imaging-guided evaluation of occult submucosal lesions.

An additional consideration is the interpretation of p16 immunohistochemistry. Strong diffuse p16 expression is widely used as a surrogate marker for HPV-associated oropharyngeal squamous cell carcinoma [14,15], which has distinct staging and prognostic implications [15,16]. However, p16 expression is not entirely specific for HPV-driven squamous malignancy and may also occur in salivary-gland-type tumors [14,17]. In the present case, approximately 70% of the tumor cells showed strong diffuse p16 positivity, but this finding was not interpreted independently. The characteristic admixture of mucous, intermediate, and epidermoid cells in cystic and solid architectural patterns supported MEC. Although HPV-specific testing was not performed, the characteristic morphology and absence of conventional squamous differentiation supported a salivary gland origin.

Molecular testing can provide additional diagnostic support. MECT1–MAML2 fusion is reported in a substantial proportion of low- and intermediate-grade MECs and has been associated with more favorable clinical behavior [18,19]. Nevertheless, absence of detectable MAML2 rearrangement does not exclude MEC [18,19]. In our patient, MAML2 rearrangement was not detected despite characteristic histomorphology. Molecular findings should therefore be interpreted as ancillary evidence rather than as standalone diagnostic criteria [18,19].

The differential diagnosis in this setting should be approached by integrating radiologic, histopathologic, and ancillary findings. Radiographically, an occult oropharyngeal squamous cell carcinoma is an important consideration in a patient presenting with cervical nodal metastasis and subtle tonsillar asymmetry [1,2,6]. Histopathologically, the presence of mucous, intermediate, and epidermoid cells in characteristic cystic and solid architectural patterns supports MEC and helps distinguish it from conventional squamous cell carcinoma and other salivary-gland-type malignancies [7,8]. Strong p16 positivity should not override these morphologic findings, because p16 expression is not entirely specific for HPV-associated squamous cell carcinoma [14,17]. Similarly, although detection of a MAML2 rearrangement can support MEC, its absence does not exclude the diagnosis [18,19]. Thus, the final diagnosis should rest primarily on characteristic morphology, with imaging, immunohistochemistry, and molecular testing serving complementary roles.

**Figure 7:**
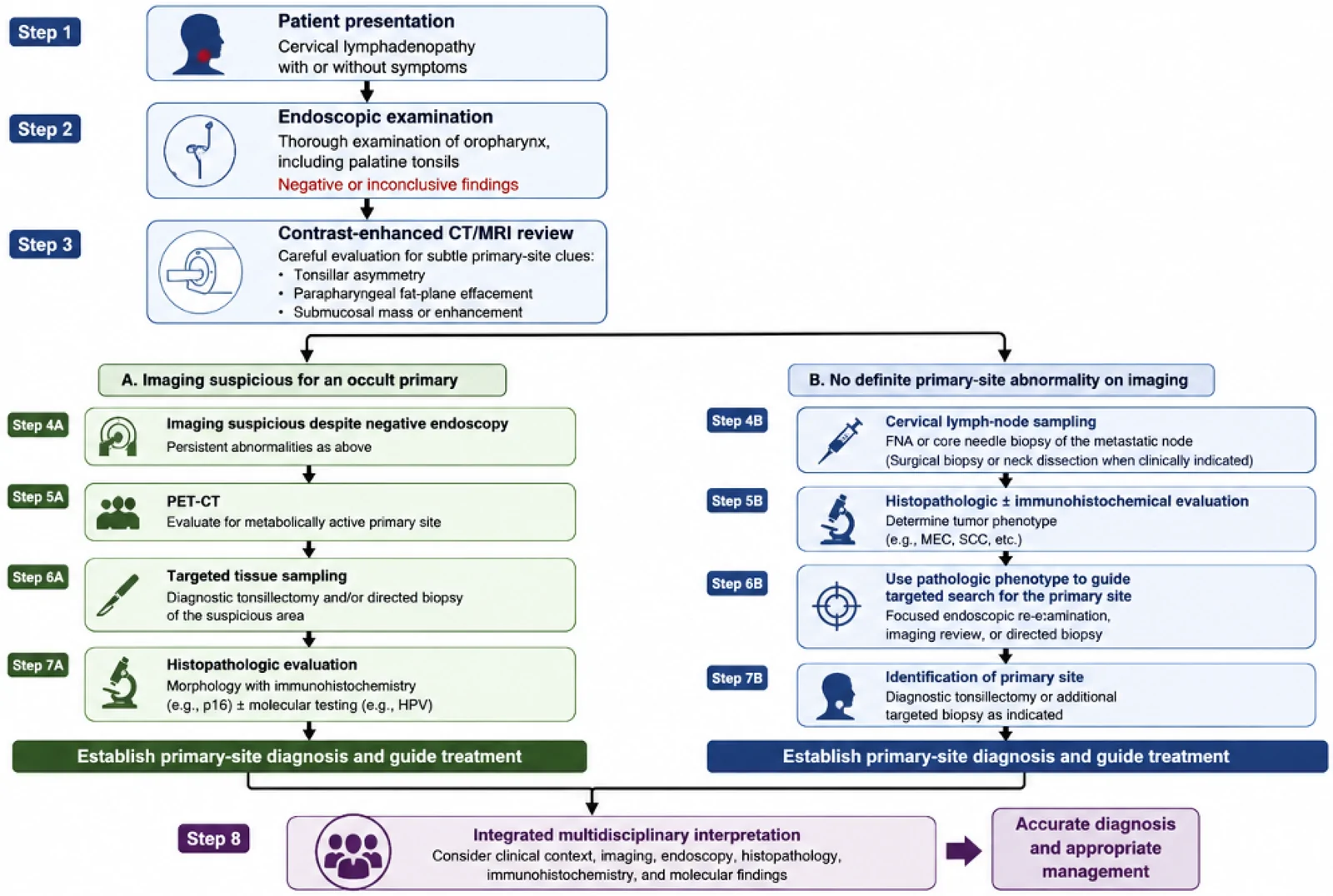
Proposed diagnostic framework for the evaluation of occult tonsillar or oropharyngeal malignancy in patients presenting with cervical lymphadenopathy and negative endoscopic findings. Persistent imaging abnormalities, including tonsillar asymmetry and parapharyngeal fat-plane effacement, should prompt targeted evaluation despite inconclusive mucosal examination. When no definite primary-site abnormality is identified on imaging, pathologic evaluation of cervical lymphadenopathy may help characterize the tumor and guide subsequent investigation of the primary site. Histopathologic interpretation should integrate morphologic findings with immunohistochemical and molecular results to support accurate tumor classification.

These observations support an integrated diagnostic strategy incorporating clinical examination, endoscopy, imaging, and pathologic evaluation (Figure 7). In patients with cervical lymphadenopathy and negative endoscopy, persistent imaging abnormalities such as tonsillar asymmetry or parapharyngeal fat-plane obliteration should prompt targeted biopsy, diagnostic tonsillectomy, or other directed evaluation [2,4,5,6]. When neither endoscopy nor imaging identifies a definite primary site, fine-needle aspiration or core biopsy of the cervical lymphadenopathy may characterize the tumor phenotype and guide subsequent investigation; surgical nodal sampling or neck dissection may provide additional pathologic information when clinically indicated [1,2,6].

Accurate distinction between MEC and HPV-related oropharyngeal squamous cell carcinoma is clinically relevant because these entities differ in staging and treatment considerations [14,15,16,17,20]. Recognition of MEC as a salivary-gland-type malignancy allows risk stratification and treatment planning according to salivary gland carcinoma principles [21,22]. Adverse features such as extranodal extension or close surgical margins may warrant postoperative radiotherapy or concurrent chemoradiotherapy [22,23]. Long-term surveillance is also important because salivary gland malignancies may recur or metastasize years after treatment [22]. Distinction from other salivary-gland-type neoplasms, particularly adenoid cystic carcinoma, remains important because of differences in biologic behaviors and recurrence patterns [24].

Overall, this case demonstrates that persistent radiologic abnormalities can identify an occult submucosal tonsillar malignancy despite negative endoscopic findings. Integrating imaging with morphology and appropriate ancillary testing is therefore essential for accurate tumor localization and classification.

## 4. Conclusions

This case demonstrates that occult tonsillar MEC may present as isolated cervical lymphadenopathy with negative endoscopic findings due to its submucosal growth pattern. Careful evaluation of imaging findings, particularly tonsillar asymmetry and parapharyngeal fat plane effacement, is essential for tumor localization. Furthermore, p16 positivity should be interpreted in conjunction with morphologic and clinicopathologic findings rather than as an isolated diagnostic marker [14,17]. A structured diagnostic approach incorporating imaging–endoscopy correlation and histopathologic confirmation is critical to achieving an accurate diagnosis and guiding appropriate management [2,4,5,6].

## Figures and Tables

**Table 1:** Published cases of palatine tonsillar mucoepidermoid carcinoma and associated diagnostic features.

Study	Presentation	Endoscopy	Imaging	Pathology	Diagnostic Lesson
Jarvis 2013 [10]	Level II neck mass	Normal	Cervical node only on CT	High-grade MEC	Occult tonsillar MEC may present as cervical metastasis despite normal endoscopy
Teixeira 2015 [13]	Dysphagia, sore throat, neck swelling	Tonsillar ulcer	Tonsillar mass	MEC, p16 negative	Histopathology is essential because tonsillar MEC may mimic common tonsillar malignancies
Goh 2017 [9]	Blood in sputum	Tonsillar lesion	Exophytic tonsillar mass	Spindle-cell MEC, CRTC1-MAML2-positive	Molecular testing may assist diagnosis of unusual variants
Karkuzhali 2017 [11]	Throat pain, dysphagia	Enlarged tonsil	NR	Low-grade MEC	Clinical presentation may resemble benign tonsillar disease
Talani 2023 [12]	Level IIa neck mass	Normal	Tonsillar enhancement on CT	High-grade MEC	Normal endoscopy does not exclude occult tonsillar MEC
Present case	Isolated cervical lymphadenopathy	Negative	CT asymmetry + PET uptake	Low-grade MEC, p16+, MAML2−	Imaging–endoscopy discordance may reveal occult submucosal tonsillar MEC

## Data Availability

The original contributions presented in this study are included in the article. Further inquiries can be directed to the corresponding authors.

## References

[1] Cianchetti M., Mancuso A.A., Amdur R.J., Werning J.W., Kirwan J., Morris C.G., Mendenhall W.M. (2009). Diagnostic evaluation of squamous cell carcinoma metastatic to cervical lymph nodes from an unknown head and neck primary site. Laryngoscope.

[2] Waltonen J.D., Ozer E., Hall N.C., Schuller D.E., Agrawal A. (2009). Metastatic carcinoma of the neck of unknown primary origin: Evolution and efficacy of the modern workup. Arch. Otolaryngol. Head Neck Surg..

[3] Rusthoven K.E., Koshy M., Paulino A.C. (2004). The role of fluorodeoxyglucose positron emission tomography in cervical lymph node metastases from an unknown primary tumor. Cancer.

[4] Di Maio P., Iocca O., De Virgilio A., Ferreli F., Cristalli G., Pellini R., Golusinski P., Ricci G., Spriano G. (2019). Role of palatine tonsillectomy in the diagnostic workup of head and neck squamous cell carcinoma of unknown primary origin: A systematic review and meta-analysis. Head Neck.

[5] Koch W.M., Bhatti N., Williams M.F., Eisele D.W. (2001). Oncologic rationale for bilateral tonsillectomy in head and neck squamous cell carcinoma of unknown primary source. Otolaryngol. Head Neck Surg..

[6] Maghami E., Ismaila N., Alvarez A., Chernock R., Duvvuri U., Geiger J., Gross N., Haughey B., Paul D., Rodriguez C. (2020). Diagnosis and Management of Squamous Cell Carcinoma of Unknown Primary in the Head and Neck: ASCO Guideline. J. Clin. Oncol..

[7] El-Naggar A.K., Chan J.K.C., Grandis J.R., Takata T., Slootweg P.J. (2017). WHO Classification of Head and Neck Tumours.

[8] Luna M.A. (2006). Salivary mucoepidermoid carcinoma: Revisited. Adv. Anat. Pathol..

[9] Goh G.H., Lim C.M., Vanacek T., Michal M., Petersson F. (2017). Spindle Cell Mucoepidermoid Carcinoma of the Palatine Tonsil with CRTC1-MAML2 Fusion Transcript: Report of a Rare Case in a 17-Year-Old Boy and a Review of the Literature. Int. J. Surg. Pathol..

[10] Jarvis S.J., Giangrande V., Brennan P.A. (2013). Mucoepidermoid carcinoma of the tonsil: A very rare presentation. Acta Otorhinolaryngol. Ital..

[11] Karkuzhali P., Sahoo B. (2017). Mucoepidermoid carcinoma of the palatine tonsil: A case report and review of the literature. Int. J. Otorhinolaryngol. Head Neck Surg..

[12] Talani C., Frånlund K., Unguras C. (2023). A rare case of tonsillar mucoepidermoid carcinoma. Acta Oto-Laryngol. Case Rep..

[13] Teixeira L.N., Montalli V.A., Teixeira L.C., Passador-Santos F., Soares A.B., de Araujo V.C. (2015). Mucoepidermoid Carcinoma of the Palatine Tonsil. Case Rep. Oncol. Med..

[14] Lewis J.S., Thorstad W.L., Chernock R.D., Haughey B.H., Yip J.H., Zhang Q., El-Mofty S.K. (2010). p16 positive oropharyngeal squamous cell carcinoma:an entity with a favorable prognosis regardless of tumor HPV status. Am. J. Surg. Pathol..

[15] Rischin D., Young R.J., Fisher R., Fox S.B., Le Q.T., Peters L.J., Solomon B., Choi J., O’Sullivan B., Kenny L.M. (2010). Prognostic significance of p16INK4A and human papillomavirus in patients with oropharyngeal cancer treated on TROG 02.02 phase III trial. J. Clin. Oncol..

[16] Fakhry C., Westra W.H., Li S., Cmelak A., Ridge J.A., Pinto H., Forastiere A., Gillison M.L. (2008). Improved survival of patients with human papillomavirus-positive head and neck squamous cell carcinoma in a prospective clinical trial. J. Natl. Cancer Inst..

[17] Brunner M., Koperek O., Wrba F., Erovic B.M., Heiduschka G., Schoppper C., Thurnher D. (2012). HPV infection and p16 expression in carcinomas of the minor salivary glands. Eur. Arch. Otorhinolaryngol..

[18] Behboudi A., Enlund F., Winnes M., Andren Y., Nordkvist A., Leivo I., Flaberg E., Szekely L., Makitie A., Grenman R. (2006). Molecular classification of mucoepidermoid carcinomas-prognostic significance of the MECT1-MAML2 fusion oncogene. Genes Chromosom. Cancer.

[19] Okabe M., Miyabe S., Nagatsuka H., Terada A., Hanai N., Yokoi M., Shimozato K., Eimoto T., Nakamura S., Nagai N. (2006). MECT1-MAML2 fusion transcript defines a favorable subset of mucoepidermoid carcinoma. Clin. Cancer Res..

[20] D’Souza G., Kreimer A.R., Viscidi R., Pawlita M., Fakhry C., Koch W.M., Westra W.H., Gillison M.L. (2007). Case-control study of human papillomavirus and oropharyngeal cancer. N. Engl. J. Med..

[21] Seethala R.R. (2009). An update on grading of salivary gland carcinomas. Head Neck Pathol..

[22] Terhaard C.H., Lubsen H., Van der Tweel I., Hilgers F.J., Eijkenboom W.M., Marres H.A., Tjho-Heslinga R.E., de Jong J.M., Roodenburg J.L., Dutch H. (2004). Salivary gland carcinoma: Independent prognostic factors for locoregional control, distant metastases, and overall survival: Results of the Dutch head and neck oncology cooperative group. Head Neck.

[23] Bernier J., Domenge C., Ozsahin M., Matuszewska K., Lefebvre J.L., Greiner R.H., Giralt J., Maingon P., Rolland F., Bolla M. (2004). Postoperative irradiation with or without concomitant chemotherapy for locally advanced head and neck cancer. N. Engl. J. Med..

[24] Coca-Pelaz A., Rodrigo J.P., Bradley P.J., Vander Poorten V., Triantafyllou A., Hunt J.L., Strojan P., Rinaldo A., Haigentz M., Takes R.P. (2015). Adenoid cystic carcinoma of the head and neck—An update. Oral Oncol..

